# Selective separation of chlorophyll-a using recyclable hybrids based on Zn-MOF@cellulosic fibers

**DOI:** 10.1038/s41598-023-42151-9

**Published:** 2023-09-14

**Authors:** Hossam E. Emam, Hanan B. Ahmed, Mahmoud El-Shahat, Hassan Abdel-Gawad, Reda M. Abdelhameed

**Affiliations:** 1 Department of Pretreatment and Finishing of Cellulosic Based Textiles, Textile Research and Technology Institute, National Research Centre, Scopus Affiliation ID 60014618, 33 EL Buhouth St., Dokki, Giza, 12622 Egypt; 2 Chemistry Department, Faculty of Science, Helwan University, Ain-Helwan, Cairo, 11795 Egypt; 3 Photochemistry Department, Chemical Industries Research Institute, National Research Centre, Scopus Affiliation ID 60014618, 33 EL Buhouth St., Dokki, Giza, 12622 Egypt; 4 Applied Organic Chemistry Department, Chemical Industries Research Institute, National Research Centre, Scopus Affiliation ID 60014618, 33 EL Buhouth St., Dokki, Giza, 12622 Egypt

**Keywords:** Green chemistry, Materials chemistry

## Abstract

Chlorophyll-a as pigments, exist in the green organelles for plants that act in photosynthesis. Different studies were considered with demonstration of an effective separation technique of Chlorophyll-a without decomposition; however, the reported methods were disadvantageous with expensiveness and low quantum yield. The current work uniquely represents an investigative method for the separation of Chlorophyll-a from spinach extract using cellulosic hybrids based on ZIF-8@cellulosic fibers (Zn-zeolitic imidazolate frameworks@cellulosic fibers) as a cost effective and recyclable absorbents. To obtain hybrids, ZIF-8 was in-situ prepared over the cellulosic fibers (bamboo, modal and cotton). The untreated and treated fibers were well characterized via FTIR, SEM, EDX, XRD, in order to approve the successive impregnation of ZIF-8. Whereas, the microscopic images showed that, microcrystalline ZIF-8 rods with length of 1.3–4.4 µm were grown over the cellulosic fibers. The obtained hybrids and the untreated fibers were exploited in the separation of Chlorophyll-a via the adsorption/desorption process. The chlorophyll-adsorption was followed Langmuir isotherm and pseudo-second order model. The Langmuir maximum capacities of Chlorophyll-a onto hybrids were followed the order of ZIF-8@cotton (583.6 mg/g) > ZIF-8@modal (561.3 mg/g) > ZIF-8@bamboo (528.7 mg/g). After incorporation of ZIF-8, the maximum adsorption capacities of cellulosic fibers were enhanced by 1.4–1.9 times. Adsorption of chlorophyll onto the applied hybrids was lowered by 27–28%, after five repetitive washing cycles. The data summarized that; chlorophyll was effectively separated by the synthesized ZIF-8@cellulosic fibers hybrids, whereas, the prepared hybrids showed good reusability for application on wider scaled purposes.

## Introduction

Chlorophyll-a are pigments exist in green organelles for plants^1^ to be mainly responsible for processing the photosynthesis. The entire composition of chlorophyll-a and b were investigated by Fischer and Wenderoth^2^. Mainly the difference between chlorophyll-a and b is their role in photosynthetic process, whereas, chlorophyll-a is the principal pigment that is involved in the photosynthetic process, while, chlorophyll b is the accessorial pigment, that act in collection of energy for passing into chlorophyll-a^3^. Both of chlorophyll-a and b are composed of chlorin ring, whereas, 4 nitrogen atoms are surrounding a Mg^2+^ ions, and different side chains and hydrocarbon tails are also bonded to the chlorin ring, however, C7 in chlorin ring is bonded to CH_3_ group in case of chlorophyll-a, but, in case of chlorophyll b, C7 position of the chlorin ring is attached to CHO group^4^. The purpose for separation of chlorophyll is to help in capturing a bit more of the spectra, plants have accessorial pigments known as carotenoids that could reflect the yellow, orange, and red light, absorbing a portion of the green light of spectrum, however, chlorophyll-a tends to mask the most of these pigments in plants, so to see these pigments, an effective method for their separation is considerably required.

Tswett^5^ was reported the first successful separation technique for chlorophyll-a via the modern chromatography. Different chromatographic techniques like conventional column^6^, paper^7^, high-performance liquid chromatography^8^ and thin-layer^9^ were investigated and applied for the analysis and separation of Chlorophyll-a. In addition to the susceptibility of chlorophyll-a to some of chemical transformation reactions that might be occurred through the extraction, the as-mentioned techniques were disadvantageous with low quantum yields. In recent years^10^, the counter-current chromatography (CCC) technique is exploited for separation of chlorophyll-a from spinach leaves.

It must be considerably taken in mind that; it is essential to exploit separation techniques with minimum effect of decomposition for the extractants. Adsorption is demonstrated as a favorable technique of separation, as it can be proceeded at ambient condition to be advantageously characterized with cost effectiveness^11–13^. Various approaches were concerned with exploiting the membrane and sorption methods for extraction and purification^14–17^. Activated carbon and weakly polar or nonpolar porous polymers were ascribed as the most commonly applicable adsorbents^14,18–21^. Considerable attention must be taken in account within processing the adsorption technique for the granular size, pore and surface area of such adsorbents^22^, in addition to the reaction temperature^23,24^. Numerous researching reports also confirmed that the composition of the adsorbents could be significantly affect the adsorption performance^14,25,26^. It was also reported that, the adsorption could be improved through $\pi$–$\pi$ (stacking) and Van der Waals interactions with the aromatic moieties of the adsorbents^26,27^.

Metal–organic frameworks (MOFs) are ascribed as a highly applicable microporous inorganic– organic coordinative polymeric matrices that are advantageous, due to, low density, tunable constructions and high surface area. These superiorly applicable matrices exhibit high potency in the adsorption^28–31^, hydrogen storage^32–34^, and catalysis^35–38^. Zeolitic imidazolate frameworks (ZIFs) as one of the highly applicable MOFs, are porous matrices that contain divalent metal and coordinately bonded with imidazole ligand. ZIF exhibits specific characters like high thermal stability compared to the other zeolites^39^.

ZIFs composed of cations such as zinc or cobalt that are coordinately bonded with nitrogen atoms by di-topic imidazolate moieties (im^−^) to give up a highly stabilized framework^28,40–42^. Each zinc cation is coordinatively bonded to four imidazolate moieties, whereas, the building units [Zn^2+^ (im^−^)_2_] seem to look like silicon dioxide tetrahedron in zeolites, with bond angle of 145° for zinc–imidazolate–zinc that is closer to that of silicon–oxygen–silicon. Guest-free ZIFs are advantageously characterized with high pore volume and surface area as classical metal organic frameworks^39,41–43^. Attributing to these advantages, ZIFs exhibit the great potentiality for the gas storing and separation^28,44–49^.

ZIF-8 as a type of Metal–organic frameworks (MOFs) is categorized as a tremendous porous coordination polymeric matrix that is successfully exploited in many purposes; attributing to the controllability of pores and high specific surface area. ZIF-8 is one type of ZIFs that considerably attracted the attention of researchers owing to its SOD (Sodalite framework) of crystal topography, with pore size of 11.6 Å^50^. ZIF-8 has Lewis acid–base characters, as Lewis acid is acquired from Zn^2+^, whereas Lewis base from nitrogen atoms in the imidazole moieties. This explains that ZIF-8 has potency as a heterogeneous catalyst, as it was reported to be applicable as a catalyst in Knoevenagel reaction^51,52^. Some approaches were studied the modification of ZIF-8 in order to enhance their characters and applications. Li et al.^53^ exploited nickel nanoparticles and ZIF-8 to improve the catalytic affinity in the Ammonia boran hydrolysis. Zahmarikan et al.^54^ studied the combination of Iridium nanoparticles and ZIF-8 to accelerate the rate of cyclohexane hydrogenation.

Consequently, the current study concerned with the separation of chlorophyll from spinach extract by using the synthesized ZIF-8@cellulosic fibers hybrid. The point of novelty in the current approach is to investigate the affinity of different cellulosic fibers to act as a supporting template for uploading ZIF-8 in order to be easily applicable as recyclable hybrid for separation of Chlorophyll-a. Firstly, ZIF-8 was in-situ synthesized over different cellulosic fibers (bamboo, modal and cotton) to obtain ZIF-8@cellulosic fibers hybrids. The prepared hybrids were characterized by different characterization tools including; scanning electron microscope, X-ray diffraction and infrared spectroscopy. Separation of Chlorophyll-a from spinach extract was systematically studied by using the obtained hybrids. Adsorption kinetic and adsorption isotherm of Chlorophyll-a were both investigated. Chlorophyll-a desorption as well as recyclability of the applied hybrids were studied.

## Results and discussion

### Characterization of ZIF-8@cellulosic fibers hybrids

**Figure 1:**
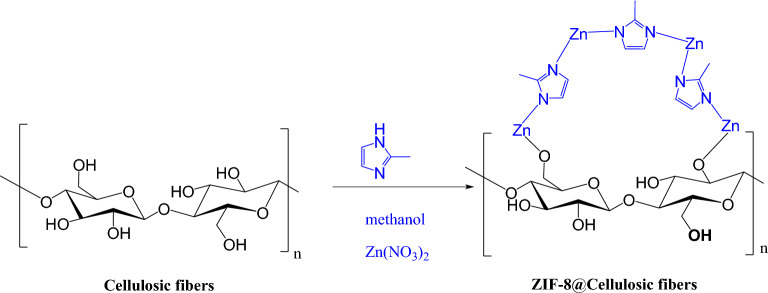
Synthesize scheme of ZIFs-8@cellulosic fibers hybrid.

ZIF-8@cellulosic fibers hybrids were obtained by in situ preparation of ZIF-8 in presence of the cellulosic fibers. ZIF-8 was synthesized and directly incorporated within the fibers as presented in Fig. 1. The cellulosic fibers were interacted with the ZIF-8 via formation bonds between hydroxylic groups of cellulose and zinc in ZIF-8. The hydrogen atoms in hydroxyl groups were substituted by zinc forming ZIF-8@cellulosic fibers hybrids with Zn–O–C bridge. Moreover, the un-substituted hydroxyl groups in fibers may form coordination and/or hydrogen bonding with –NH and/or Zn in ZIF-8, respectively^55,56^.

### SEM

**Figure 2:**
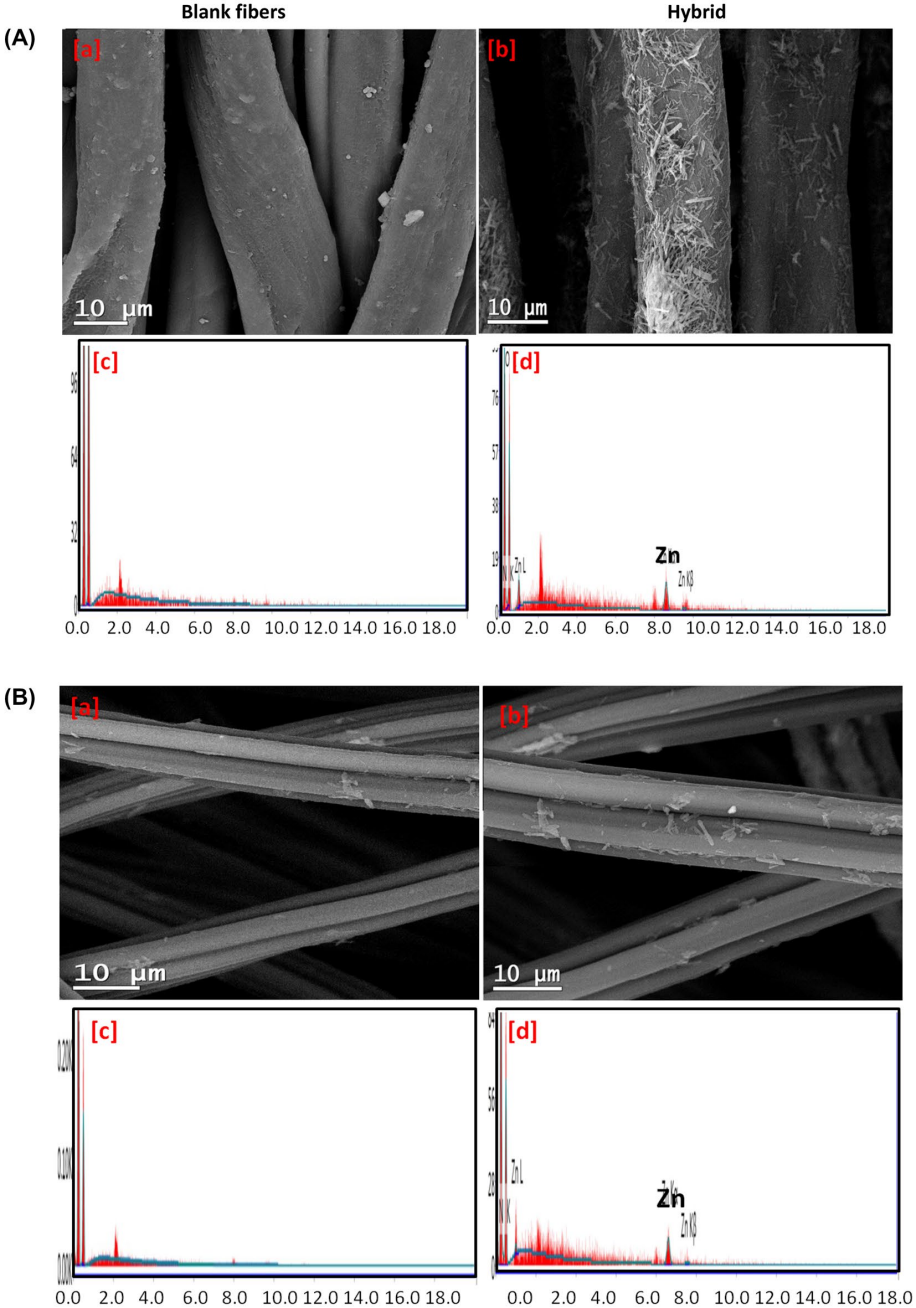

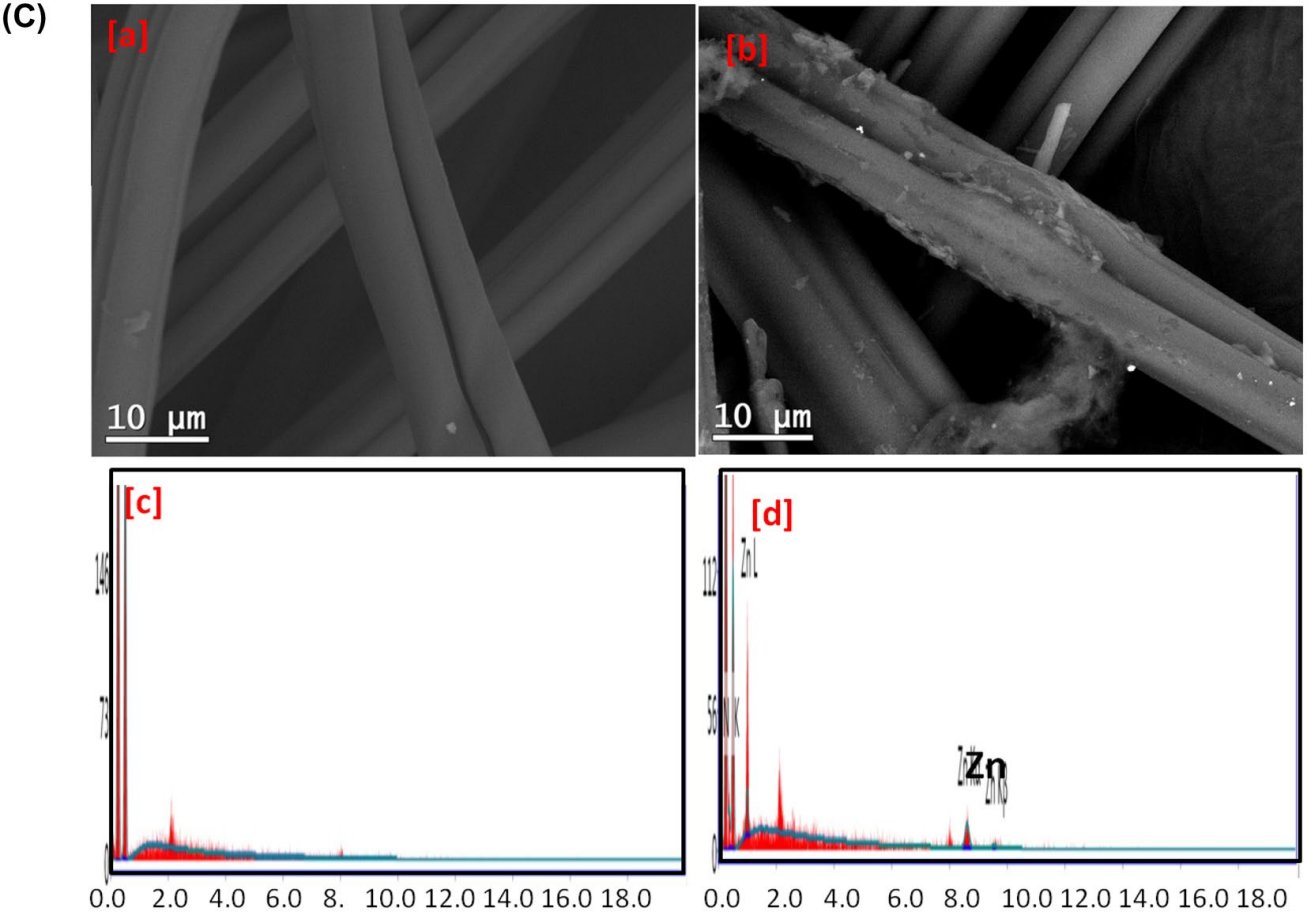
Micrographs & EDX analysis for the obtained ZIFs-8@cellulosic fibers hybrid; (**A**) ZIF-8@bamboo fibers, (**B**) ZIF-8@modal fibers and (**C**) ZIF-8@cotton fibers.

The morphological and the surface characteristics of the obtained ZIF-8@cellulosic fibers hybrids (ZIF-8@bamboo, ZIF-8@modal and ZIF-8@cotton) were presented as micrographs in Fig. 2. As a comparison, the micrographs and elemental analysis spectra for untreated fibers (bamboo, modal and cotton) were also shown. Smooth and clean surface of untreated cellulosic fibers was observed and the elemental analysis showed only the signals of carbon and oxygen which are related to cellulose backbone. For the synthesized hybrids, microcrystalline ZIF-8 particles were obviously grown over the cellulosic fibers. Compared to bamboo and modal fibers, the cotton fibers were densely covered with ZIF-8 particles which may be due to its higher reactivity. Regardless to the nature of cellulosic fibers, ZIF-8 with rod-like structure was viewed onto the applied fibers. The length of ZIF-8 rode particles onto fibers was ranged in 1.3–4.4 µm. Based on the literature, there were different geometrical shapes for ZIFs-8 represented in hexagonal, spherical and octagonal according to the used experimental conditions^57–61^. In the present work, epitaxial growth of ZIF-8 (in rod-like structure) over the fibrils and hence similar shape for fibers was produced, as all hybrids were prepared under the same experimental conditions. The elemental analysis spectra confirmed that the all of the prepared hybrids showed the signals of nitrogen and zinc which characterized for ZIF-8, besides those of carbon and oxygen for cellulose.

### XRD

**Figure 3:**
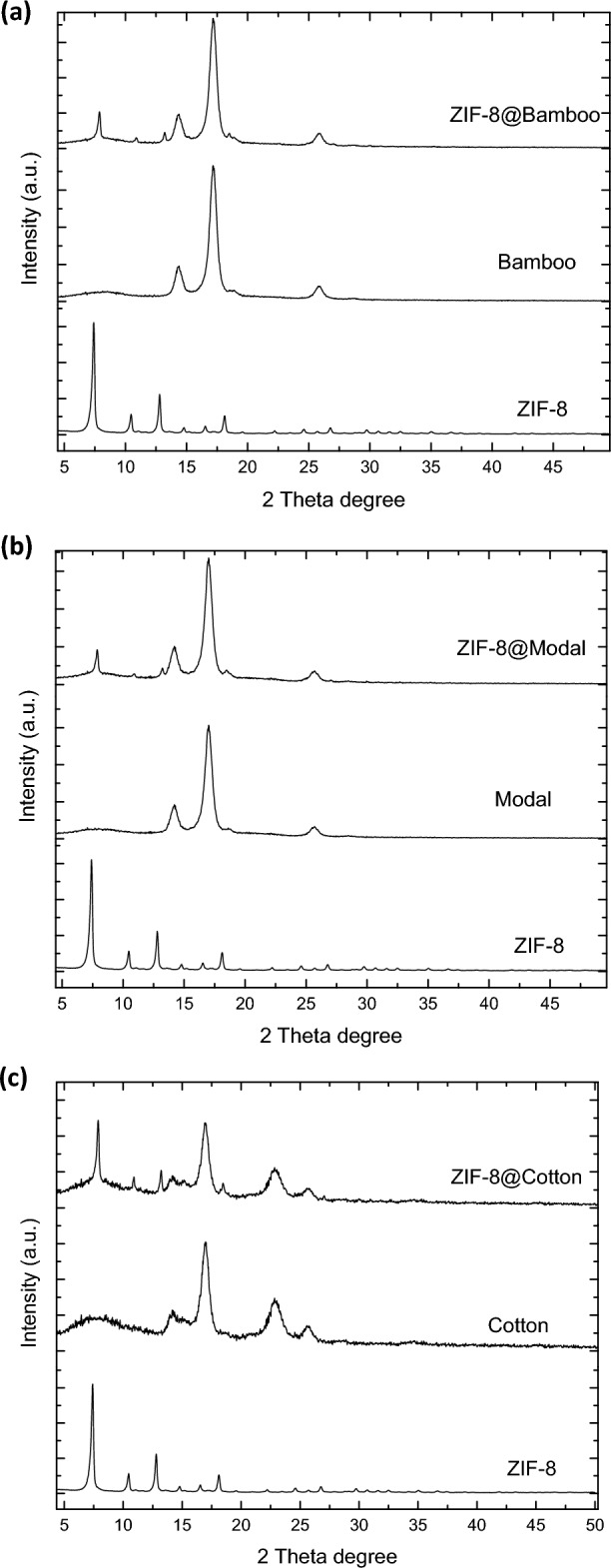
XRD for the synthesized ZIFs-8@cellulosic fibers.

Diffractograms of the synthesized hybrids and the untreated fibers were shown in Fig. 3. For the untreated bamboo and modal fibers, three diffractions were recorded at 2θ = 14.5°, 16.6° and 25.8°. While for the untreated cotton, four diffractions at 2θ = 14.4°, 16.5°, 22.8° and 25.8° were clearly observed. The recorded diffractions are characterized for the crystalline cellulose in the fibers. The four main diffractions of ZIF-8 at 2θ = 7.2°, 10.2°, 12.7° and 18.0°^57,62,63^ were appeared for all synthesized ZIF-8@cellulosic fibers hybrids beside those of cellulose. These recorded diffractions are corresponded to the crystalline lattice (110), (200), (211) and (222) for the formed ZIFs-8^58,59,63,64^. Compared to that were estimated for bamboo and modal, the intensity of ZIF-8 diffractions was high in case of cotton, due to, higher ZIF-8 amount onto fibers as viewed from micrographs. These diffractions are in harmony with the micrographs observations and further confirmed the formation of ZIF-8@cellulosic fibers hybrids.

### FTIR

**Figure 4:**
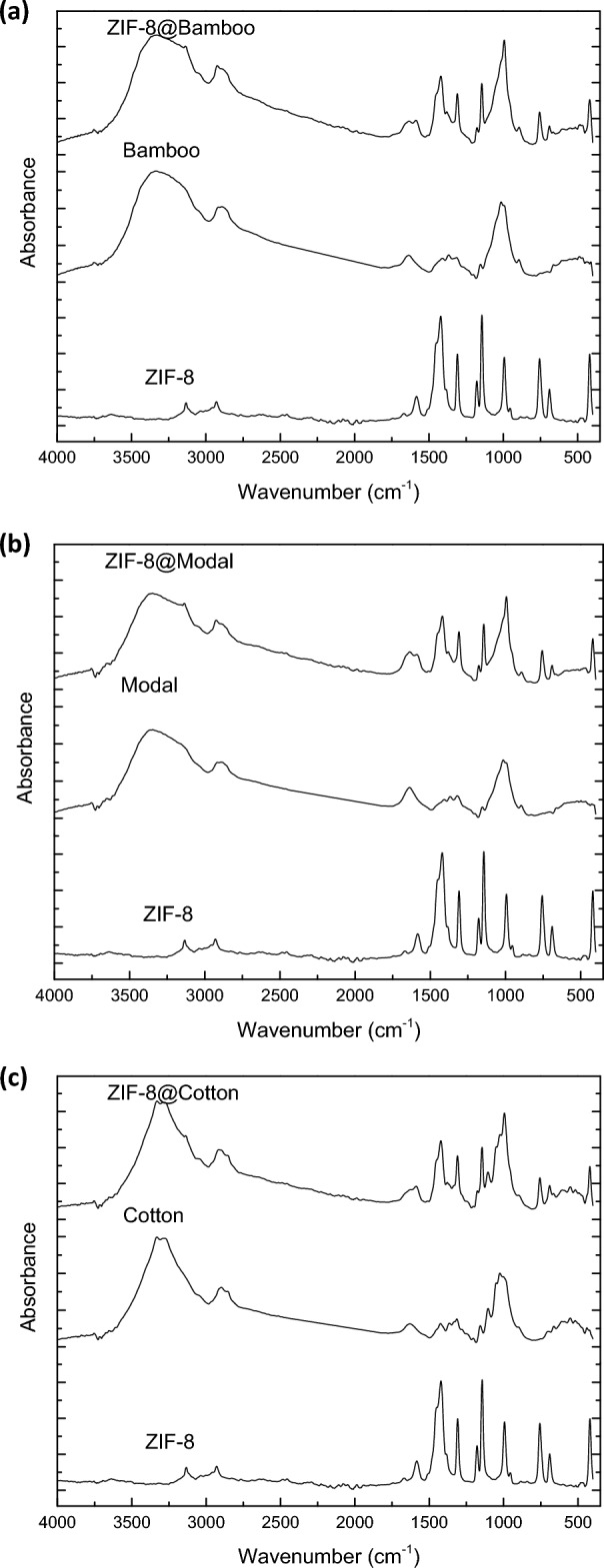
IR spectra for the synthesized ZIFs-8@cellulosic fibers.

The spectral data of the infrared were measured for the untreated fibers (bamboo, modal and cotton) and the synthesized hybrids (ZIF-8@bamboo, ZIF-8@modal and ZIF-8@cotton) and the obtained spectra were collected in Fig. 4. Regardless to the type of fibers, the untreated cellulosic fibers exhibited five main absorbance peaks at the wavenumber of 3268–3292, 2848–2866, 1604–1627, 1306–1378 and 992–998 cm^−1^ which are characterized for their cellulosic backbone^55,65^. The highlighted peaks are attributed to the vibration of hydroxyl group, asymmetric of C–H aliphatic, carbonyl group, bending of C–H and the bending of C–C, respectively. For the obtained hybrids, all the characteristics peaks of the cellulosic fibers were appeared. Besides, four new peaks were observed at 1424/1305 cm^−1^, 1165 cm^−1^ and 748 cm^−1^ which are corresponding to the amide groups, C–N, and imidazole ring for the ZIF-8, respectively^60,61^. Additionally, the absorption peaks of N–H (3182 cm^−1^), aliphatic C–H (29 cm^−1^), imidazole ring (1583 cm^−1^) for the ZIF-8 were overlapped with the absorption peaks of O–H, aliphatic C–H and C=O for cellulose. The peak of Zn–N bond was red shifted (lower wavenumber) attributing to the formation of coordination bonding with the cellulose. The observed spectral data are showed the interaction between ZIF-8 and fibers to consequently confirm the successful formation of ZIF-8@celluosic fibers hybrids.

### Separation of Chlorophyll-a

**Figure 5:**
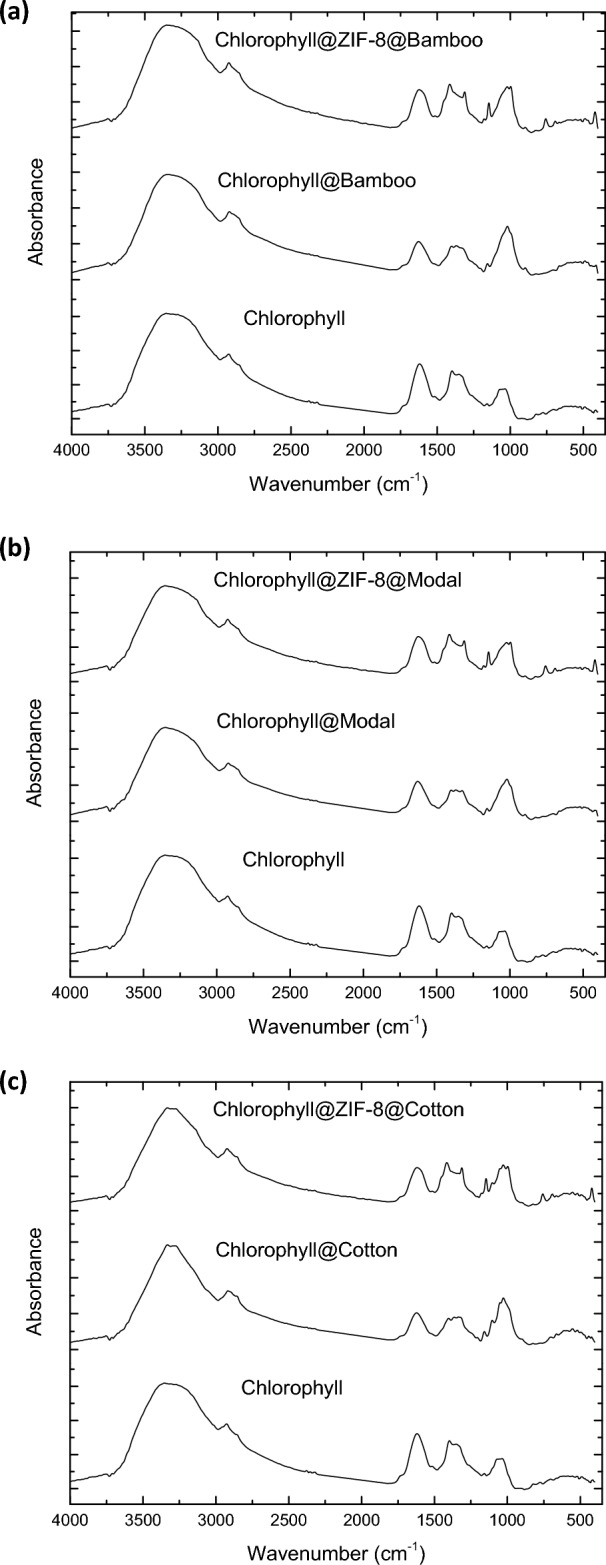
IR spectra for ZIFs-8@cellulosic fibers after chlorophyll adsorption.

**Figure 6:**
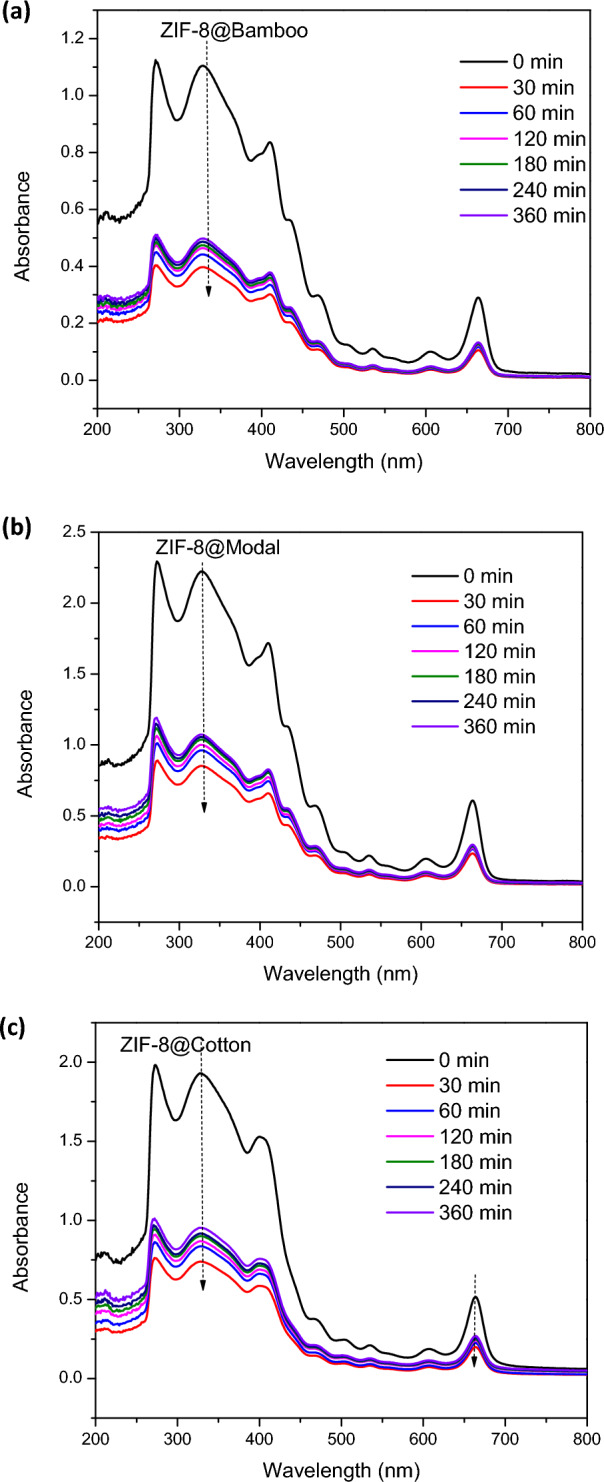
Absorbance spectra for the chlorophyll after adsorption onto the synthesized ZIFs-8@cellulosic fibers as function of time; (**a**) ZIF-8@bamboo fibers, (**b**) ZIF-8@modal fibers and (**c**) ZIF-8@cotton fibers.

**Figure 7:**
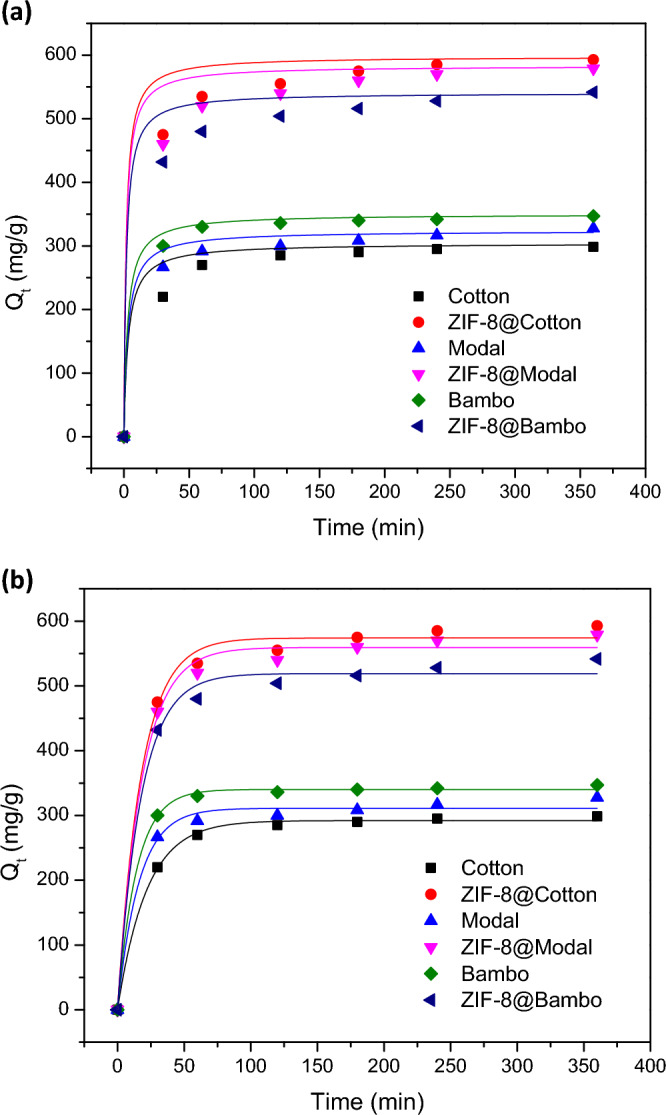
Adsorption kinetic for chlorophyll adsorption onto the synthesized ZIFs-8@cellulosic fibers; (**a**) pseudo-first order and (**b**) pseudo-second order.

The obtained hybrids (ZIF-8@bamboo, ZIF-8@modal and ZIF-8@cotton) were applied in Chlorophyll-a separation from the spinach extract. The untreated cellulosic fibers were also used in Chlorophyll-a separation for the real comparison with hybrids. Selectivity of ZIF-8@cellulosic fibers was approved via HPLC analysis (Supplementary data, Fig. S1). The separation of Chlorophyll-a was performed by adsorption onto fibers followed by separation from fiber surface through desorption process. The chlorophyll-adsorption onto the applied fibers/hybrids was studied as function of the contact time and Chlorophyll-a concentration. The absorbance spectra for the residual Chlorophyll-a in the tested solutions were exported and presented in Fig. 5. From the showed spectra, the concentration of Chlorophyll-a in the residual solution was significantly diminished by increment in the adsorption time which declares the increment in Chlorophyll-a adsorption onto the applied hybrids. The residue concentration of Chlorophyll-a was detected by estimating the absorbance peak intensity at 663 nm. Consequently, the adsorbed Chlorophyll-a amount was evaluated and sketched with the contact time as kinetics (Fig. 6, kinetic) and chlorophyll concentration as isotherm (Fig. 7).

As notified from Fig. 6, the adsorption behavior of Chlorophyll-a onto the all-applied materials (fibers and hybrids) was identical and the Chlorophyll-a adsorption was progressively increased with the adsorption time. Chlorophyll-a adsorption was rapid in the first hour and slowed down in the next 2 h. This may be demonstrated by the higher freely accessible active sites at the beginning of adsorption which were conquered and became unavailable by time. The results declared that the hybrids showed significantly higher adsorption capacities rather than the untreated cellulosic fibers. The adsorption capacity of Chlorophyll-a onto the applied fibers and hybrids was followed the order of cotton < modal < bamboo and ZIF-8@cotton ≥ ZIF-8@modal > ZIF-8@bamboo, respectively. The highest adsorption capacity for ZIF-8@cotton could be attributed to the highest loaded ZIF-8 amount on cotton fibers compared to the other fibers.

Within the first 30 min, the adsorption capacity of Chlorophyll-a onto the untreated fibers was 218, 266 and 294 mg/g for cotton, modal and bamboo fibers, respectively. While the adsorption capacities onto hybrids were considerably increased to 429, 458 and 473 mg/g for ZIF-8@cotton, ZIF-8@modal and ZIF-8@bamboo, respectively. After 360 min, Chlorophyll-a adsorption capacities were 298.6, 327.5 and 345.9 mg/g for cotton, modal and bamboo and 592.8, 578.8 and 541.7 mg/g for ZIF-8@cotton, ZIF-8@modal and ZIF-8@bamboo, respectively. After incorporation of ZIF-8 within cellulosic fibers, the adsorption capacity of Chlorophyll-a was significantly enlarged by 2, 1.8 and 1.6 times in case of bamboo, modal and cotton fibers, respectively.

In order to investigate the adsorption kinetic of Chlorophyll-a onto the applied materials (untreated fibers and hybrids), the non-linear fitting of adsorption data was implemented to pseudo-first order and pseudo-second order model (Fig. 6). All parameters of kinetic (Q_e_, K_1_, K_2_, R^2^) were summarized in Table 1 and the chi-squared test (*x*^*2*^) as fitting analysis was calculated and inserted. For the all-applied hybrids, the experimental adsorption capacities (Q_exp_) were quite similar to that theoretically estimated for the pseudo-second order model. Moreover, the calculated *x*^*2*^ values were smaller in case of pseudo-second order compared to that of pseudo-first order. Subsequently, Chlorophyll-a adsorption onto the applied ZIF@cellulosic fibers hybrids ZIFs-8 was kinetically followed to the pseudo-second order model. The estimated reaction rate (K_2_) for chlorophyll-adsorption was 1.23 × 10^–4^, 1.58 × 10^–4^ and 1.89 × 10^–4^ L/mg min for cotton, modal and bamboo and 2.14 × 10^–4^, 2.85 × 10^–4^ and 3.12 × 10^–4^ L/mg min for ZIF-8@cotton, ZIF-8@modal and ZIF-8@bamboo, respectively. This means that, the incorporation of ZIF-8 resulted in accelerating the adsorption of chlorophyll-and the fastest rate of adsorption was recorded for the ZIF-8@cotton fibers hybrid. The basis of pseudo-second order kinetic is declared that the rate of chlorophyll-adsorption onto ZIF-8@cellulosic fibers hybrids was depended on Chlorophyll-a concentration.

**Table 1:** Kinetic parameters for adsorption of chlorophyll-a on the synthesized ZIF-8@cellulosic fibers hybrids.

Samples	Q_e_exp. (mg/g)	Pseudo-first-order				Pseudo-second-order			
		Q_e_ (mg/g)	K_1_ × 10^–3^ (minute^-1^)	R^2^	χ^2^	Q_e_ (mg/g)	K_2_ × 10^–4^ (L/mg min)	R^2^	χ^2^
Cotton	298.6	292.1	45.7	0.98	701.9	304.2	1.23	0.99	21.0
ZIF-8@cotton	592.8	474.1	56.1	0.99	2428.9	597.9	2.14	0.98	248.2
Modal	327.5	311.1	62.1	0.98	278.1	323.8	1.58	0.99	117.5
ZIF-8@modal	578.8	559.4	55.1	0.96	2459.8	583.4	2.85	0.99	251.1
Bamboo	345.9	340.3	70.1	0.99	96.8	350.2	1.89	0.99	18.7
ZIF-8@bamboo	541.7	519.1	56.5	0.95	8170.9	541.1	3.12	0.98	268.9

**Figure 8:**
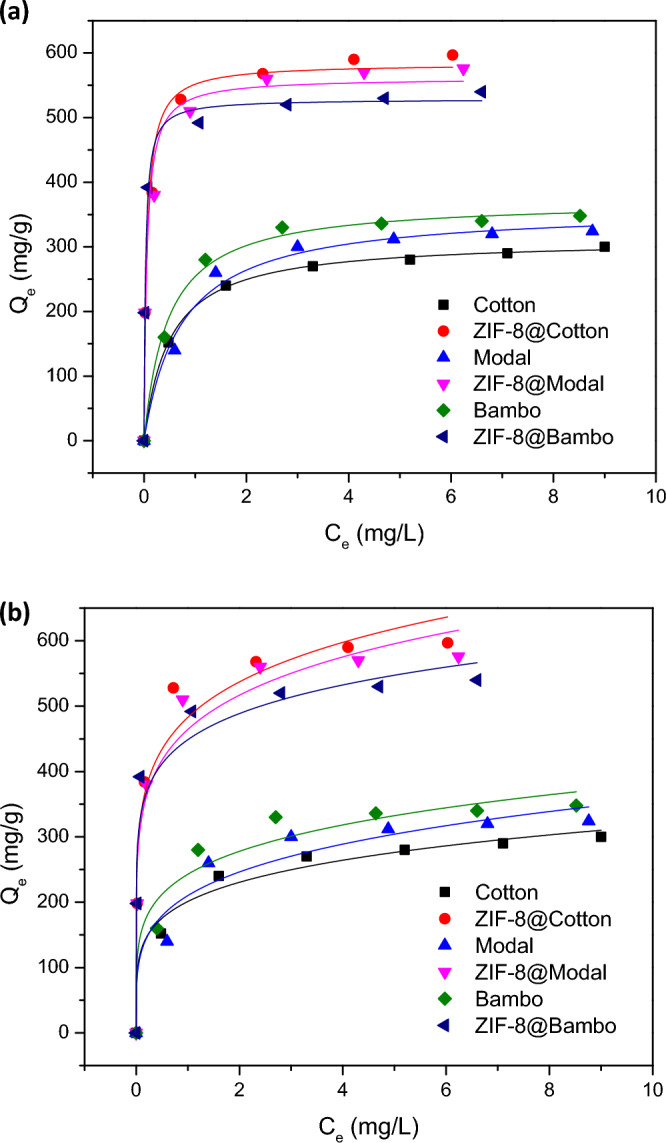
Profile of isotherm for chlorophyll adsorption onto the synthesized ZIFs-8@cellulosic fibers; (**a**) Langmuir model and (**b**) Freundlich model.

To study the adsorption isotherm, the adsorption capacity of Chlorophyll-a was drawn with the initial concentration of Chlorophyll-a (1–9 mg/L) as given in Fig. 7. It was found that, the adsorption capacity of Chlorophyll-a onto the applied materials (untreated fibers and hybrids) was gradually grown up with the initial concentration until attain the plateau at the optimal concentration of 3 mg/L. After the optimal concentration of Chlorophyll-a, marginal increment was recorded in the adsorption capacity. The isotherm for Chlorophyll-a adsorption was checked for the non-linear fitting to Langmuir and Freundlich model (Fig. 8) and the all parameters of isotherm (n, K_F_, Q_m_, K_L_, R^2^) as well as *x*^*2*^ values were all collected in Table 2. Owing to smaller values of *x*^*2*^ and higher values of R^2^, the adsorption isotherm data of Chlorophyll-a are very suitable to the Langmuir model. Consequently, the chlorophyll-adsorption onto the untreated fibers and hybrids was performed by formation of the monolayer^28,66,67^. The estimated maximum capacity of Chlorophyll-a adsorption onto cotton, modal and bamboo was 311.8, 359.8 and 371.9 mg/g, respectively, and onto ZIF-8@cotton, ZIF-8@modal and ZIF-8@bamboo was 583.6, 561.3 and 528.7 mg/g, respectively. After the incorporation of ZIF-8 within the cellulosic fibers, the estimated maximum capacities of chlorophyll-adsorption were significantly enlarged by factor of 1.4–1.9. Moreover, according to the estimated elemental analysis (Table S1), ZIF-8@cotton fibers hybrid, to exhibit the highest maximum capacity of Chlorophyll-a adsorption from spinach extract owing to the highest ZIF-8 content (8.51 ± 0.92). Whereas, cotton with the highest cellulose content compared to modal and bamboo, so as, cotton is exhibited with more of accessible functional groups that could successively bonded with more of ZIF-moieties, resulting in higher amounts of adsorbed chlorophyll. Moreover, for more affirmation, the metal content was estimated to be the highest estimated value in case of ZIF-8@cotton compared to the other hybrids.

**Table 2:** Isotherm parameters for adsorption of chlorophyll-a on the synthesized ZIF-8@cellulosic fibers hybrids.

Samples	Freundlich				Langmuir			
	*n*	K_F_	R^2^	χ^2^	Q_m_ (mg/g)	K_L_ × 10^–2^	R^2^	χ^2^
Cotton	5.1	201.1	0.98	248.4	311.8	199.7	0.99	9.9
ZIF-8@cotton	6.5	482.9	0.96	2317.0	583.6	1729.2	0.99	100.4
Modal	4.4	210.1	0.94	954.2	359.8	137.8	0.98	250.8
ZIF-8@modal	6.5	464.4	0.97	1761.3	561.3	1747.2	0.98	141.2
Bamboo	5.1	242.6	0.95	905.4	371.9	215.7	0.99	105.9
ZIF-8@bamboo	8.1	448.7	0.94	2549.8	528.7	3192.0	0.99	181.3

Chlorophyll-a adsorption onto the applied hybrids materials (untreated fibers and ZIF-8@fibers hybrids) was further affirmed by the infrared spectra for the adsorbents after the adsorption step as shown in Fig. 5. Chlorophyll exhibited five main spectral absorption peaks at 3338, 2911/2844, 1702/1604, 1383 and 1016 cm^−1^. These highlighted peaks are corresponded to the O–H group, CH_3_/CH_2_ aliphatic, C=O/C=C aromatic, C–N and C–O, respectively as recorded in literature^68–70^. After adsorption of Chlorophyll-a, the absorption peaks of C=C, C–N and C–O for Chlorophyll-a were detected for the applied fibers and hybrids which confirmed the adsorption of Chlorophyll-a onto the fibers and hybrids.

The Langmuir maximum adsorption capacity for the ZIF-8@cellulosic fibers hybrid (544–596 mg/g) was compared with that obtained in literature for several adsorbents as reported in Table 3. Adsorption of Chlorophyll-a from diverse sources including spirulina (benzene extract), rapseed oil, rice brain oil, olive oil and model oil (xylene solution) was previously studied in literature. Very low adsorption capacities of Chlorophyll-a were obtained (0.1–10.2) by applying silica gel, acid activated speiolite, acid activated montmorillonite clay, acid activated bentonite and activated earth, as adsorbent material^71–77^. Using of folded-sheet mesoporous material exhibited moderate adsorption capacity of Chlorophyll-a from spirulina and the capacity reached 292.0 mg/g^71^. Rational high Chlorophyll-a adsorption was achieved from olive oil with capacity of 472.6 mg/g functionalized mesoporous silica^78^. Based on the summarized data from literature, the all presently synthesized ZIF-8@cellulosic fibers hybrids exhibited extremely higher maximum adsorption capacities of Chlorophyll-a than the functionalized mesoporous silica. Consequently, the analogical results recommended that the synthesized hybrids can be effectively applied in the chlorophyll-adsorption from the extract.

**Table 3:** Adsorption capacities (Q_m_) of chlorophyll-a onto different adsorbents from literature.

Adsorbent	Chlorophyll source	Maximum capacity (mg/g)	Reference number
ZIF-8@cotton fibers	Spinach	583.6	Current work
Silica gel	Spirulina (benzene)	7.0	71
Acid activated sepiolite	Rapeseed oil	0.4	72
Acid-activated montmorillonite calys	Rapeseed oil	1.1	73
H_2_SO_4_-activated bentonite	Olive oil	0.1	74
H_2_SO_4_-Activated bentonite	Model oil (xylene and edible oil solution)	3.9–7.4	75
Acid-activated bentonite	Model oil (xylene solution)	9.7–10.2	76
Activated earth (Tonsil Supreme 110 FF)	Rice brain oil	1.3–1.8	77
Folded-sheet mesoporous material	Spirulina (benzene)	40.0–292.0	71
Functionalized mesoporous silica	Olive oil	90.4–472.6	78

**Figure 9:**
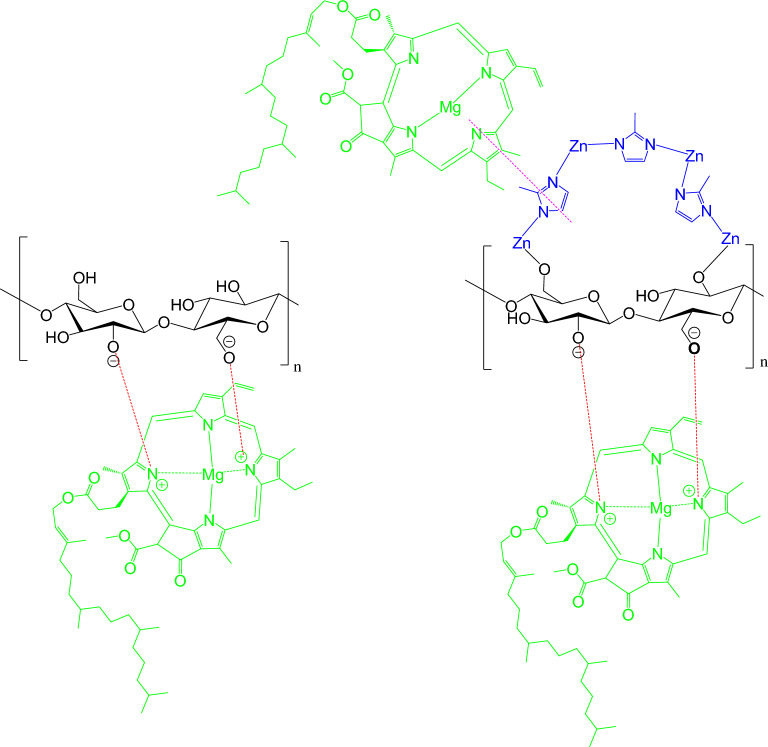
Plausible mechanism of chlorophyll adsorption onto ZIFs-8@cellulosic fibers.

The adsorption of Chlorophyll-a onto the applied cellulosic fibers (bamboo, modal and cotton) and hybrids (ZIF-8@bamboo, ZIF-8@modal and ZIF-8@cotton) was performed through physical and chemical pathways as shown in Fig. 9. In the physical pathway, Chlorophyll-a was basically deposited within the inner spaces of the fibers and/or pores of porous ZIF-8. However, the chemical pathway is the most probable rather than the physical pathway. Based on the chemical structure of Chlorophyll-a, three chemical interactions can take a place between the applied materials and Chlorophyll-a during the adsorption process. Coordination bonds may be formed between Zn of ZIF-8 in hybrids and O of Chlorophyll-a^79^. Hydrogen bonds can be formed between O and H of cellulose in fibers/hybrids and H and O in Chlorophyll-a. An electrostatic interaction occurs between the negatively charged cellulose surface and the positively charged Chlorophyll-a^80,81^. Additionally, the imidazole rings of ZIF-8 can easily form π–π interactions with the azole rings of Chlorophyll-a^82,83^. Due to its active sites (pores and functional groups), insertion of ZIF-8 in the cellulosic fibers significantly improved the Chlorophyll-a adsorption. Comparing to the other hybrids, ZIF-8@cotton fibers hybrid showed the highest adsorption capacity of Chlorophyll-a which is explained by the higher ZIF content.

### Desorption, release and recyclability

**Figure 10:**
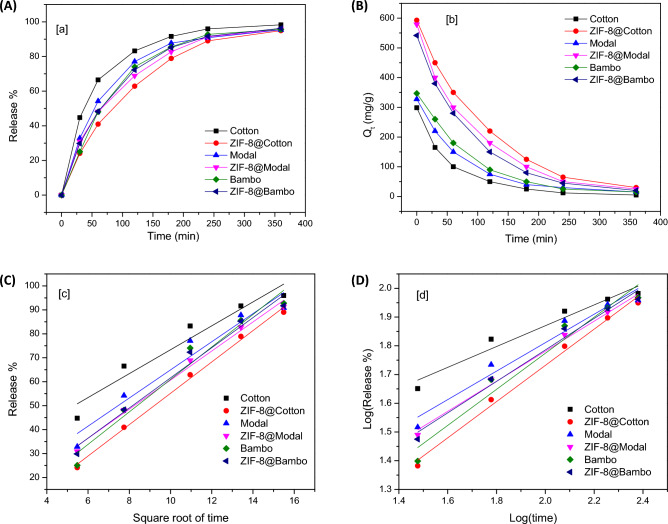
Cumulative release–time relationship of the chlorophyll from the fibers and ZIFs-8@cellulosic fibers hybrids: (**A**) release percentage, (**B**) amount of chlorophyll released, (**C**) Higuchi model for kinetic of the chlorophyll release and (**D**) Korsemeyer-Peppas model for kinetic of the chlorophyll release.

To achieve the separation of Chlorophyll-a from spinach extract, desorption of the adsorbed Chlorophyll-a was investigated. For desorption of Chlorophyll-a, the applied chlorophyll@ZIF-8@cellulosic fibers were placed in acetone at different time. UV–Vis spectra for the desorbed chlorophyll-a was mapped to be presented in supplementary data (Fig. S2). To optimize the best desorption conditions, the release of Chlorophyll-a was systematically studied with different kinetic models including Higuchi and Krosmeyer-Peppas as showed in Fig. 10.

The profile of Chlorophyll-a release from the applied hybrids was estimated by release percentage (Fig. 10A) and release amount (Fig. 10B). Regardless to the hybrids used, the release profiles were similar. Chlorophyll-a was progressively released by the time, while the release was quite fast in the first hour. The released Chlorophyll-a was high in case of hybrids rather than untreated fibers which are logically due to the higher adsorbed Chlorophyll-a onto hybrids. The data declared that, Chlorophyll-a was fully released after 6 h. Higuchi (Fig. 10C) and Korsmeyer-Peppas (Fig. 10D) as two different kinetic studies were performed for the investigation of the release of Chlorophyll-a to understand the mechanism of release. The kinetic parameters of Chlorophyll-a release was presented in Table 4. According to the Korsmeyer-Peppas, the release of Chlorophyll-a was mainly depended on the amount of chlorophyll-adsorbed. n values in Krosmeyer-Peppas are higher than 0.5, which confirmed that the release is not performed through diffusion. The data showed good linearity for Higuchi modeling which resulted in the proportional relationship between Chlorophyll-a release and surface area of hybrids.

**Table 4:** Kinetic parameters for the release of chlorophyll-a from the fibers and hybrids.

Samples	Higuchi		Korsemeyer-Peppas		
	K × 10^–2^	R^2^	n	K × 10^–2^	R^2^
Cotton	499.3	0.92	1.1	362.4	0.94
ZIF-8@cotton	654.8	0.99	0.5	630.6	0.99
Modal	587.7	0.93	0.8	495.5	0.95
ZIF-8@modal	607.2	0.99	0.7	523.1	0.99
Bamboo	677.9	0.95	0.5	627.7	0.95
ZIF-8@bamboo	629.4	0.97	0.7	547.7	0.98

**Figure 11:**
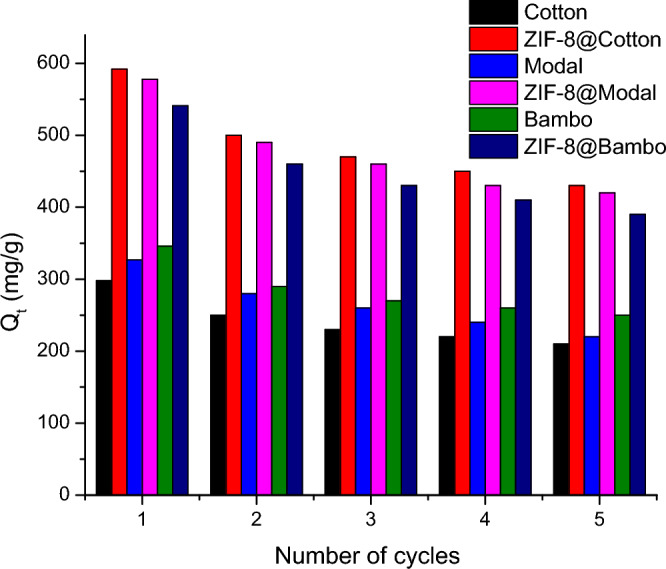
Effect of reusability for ZIFs-8 on the chlorophyll adsorption.

The kinetic of release summarized that the released (desorption) of Chlorophyll-a from hybrids is depended on the amount of adsorbed chlorophyll-and is functional of the surface area. For completely release of Chlorophyll-a from the applied adsorbents, 6 h is required. Consequently, for study the recyclability, the applied adsorbents were immersed in acetone for 6 h. The reusability of the applied ZIF-8@cellulosic fibers hybrids was studied through investigation of the recyclability to optimize the reusing of hybrids in adsorption of Chlorophyll-a several cycles. After Chlorophyll-a desorption, the applied hybrids were submerged in the acetone for 6 h to complete Chlorophyll-a removal. After desorption, the applied adsorbents (untreated fibers and hybrids) were air dried and then reuse in the higher adsorption cycles. The reusability was investigated up to 5 cycles by repeating the sorption/desorption process for 5 repetitive cycles. According to Fig. 11, the adsorption capacities of Chlorophyll-a onto the applied adsorbents were reduced after recycling, whatever the used adsorbent. Within 5 reusing cycles, the adsorption capacity of Chlorophyll-a was decreased from 541.1 to 389.0 mg/g for ZIF-8@bamboo, from 583.4 to 420.8 mg/g for ZIF-8@modal and from 597.9 to 431.3 mg/g for ZIF-8@cotton. In case of untreated cellulosic fibers, the adsorption capacities were diminished from 304.2–350.2 mg/g to 211.6–249.7 mg/g after 5 cycles. The data summarized that, the adsorption of Chlorophyll-a onto untreated cellulosic fibers and ZIF-8@cellulosic fibers was lowered by 29–33% and 27–28%, respectively after 5 consecutive cycles. Moreover, the surface area and metal content before and after repeated adsorption cycles were estimated (Table S2), whereas, the surface area and metal content is maximized in case of ZIF-8@cotton even after four adsorption cycles to be 558 m^2^/g (before adsorption 610 m^2^/g) and 7.19 mg/g (before adsorption 8.51 mg/g), respectively. This approves that, the synthesized ZIF-8@cellulosic fibers hybrids exhibited superior reusability in the Chlorophyll-a adsorption and consequently the hybrids could be applied in large scale for separation of Chlorophyll-a.

## Experimental section

### Materials

Zinc nitrate hexahydrate (Zn (NO_3_)_2_⋅6H_2_O, CAS No.: 10196-18-6, 98%), 2-methylimidazole (C_4_H_6_N_2_, CAS No.: 693-98-1, 99%), Methanol (CH_3_OH, HPLC, CAS No.: 67-56-1, ≥ 99.9%), Ethanol (CH_3_CH_2_OH, CAS No.: 64-17-5, 99.8%) and Acetone (CH_3_CH_3_CO, ACS reagent, CAS No.: 67-64-1, ≥ 99.5%) were all purchased from Sigma-Aldrich and were used as received.

Three different cellulosic fibers were used namely; bamboo, modal and cotton. The bamboo fibers with linear density of 1.3/1.4 denier/dtex and average length of 38.0 mm were purchased from East Asia Textile Technology Limited—Shanghai, China. While, the modal fibers with linear density of 1.3/1.5 denier/dtex and average length of 36.0 mm were supplied as gift samples from Lenzing Fibers Grims by Limited—United Kingdom. Cotton fibers with linear density of 2.3/2.6 denier/dtex and average length 33.8 mm were supported as gift sample from Misr Company for Spinning and Weaving, El-Mahalla El-Kobra–Egypt.

### Processes

#### Preparation of hybrids

The hydrides of ZIF-8@cellulosic fibers (ZIF-8@bamboo, ZIF-8@modal and ZIF-8@cotton) were synthesized via in-situ technique, while, the crystalline ZIF-8 were epitaxial formed over the fibers. In brief, 2-methylimidazole (3.3 g) and zinc nitrate (1.5 g) were separately dispersed in 100 mL of ethanol under continuous stirring at room temperature for one hour. Subsequently, 1 g of the cellulosic fibers (bamboo, modal and cotton) was gently submerged in the solution of zinc ions under the continuous stirring. After 1 day, the solution of ligand (2-methylimidazole) was sweetly added onto the mixture. The reaction of mixture was further continued for one hour. The hybrids (ZIF-8@cellulosic fibers) were removed, gently washed with methanol and finally dried at 60 °C under vacuum prior to characterization and applications. The obtained hybrids were labeled as ZIF-8@bamboo, ZIF-8@modal and ZIF-8@cotton fibers.

#### Extraction of Chlorophyll-a

Spinach as Chlorophyll-a source, was picked from the market and cut into small slices. Chlorophyll-a was extracted from the spinach leaves using sonication and maceration at room temperature. The spinach leaves were submerged in 80% ethanol/water mixture using solid/liquid ratio was 1/2 for 1 h. The plant cellular membrane was disrupted by action of sonication and using nonpolar solvents that is specified for the extraction of Chlorophyll-a. The extracted Chlorophyll-a with dark green color were filtered and centrifuged to remove the coarse particles. For analyzing of Chlorophyll-a, UV–Visible spectrophotometer (Shimadzu, Japan, Model UV-160A) was used.

### Characterization

The morphological features of the synthesized hybrids (ZIF-8@bamboo, ZIF-8@modal and ZIF-8@cotton) were investigated under high-resolution scanning electron microscope (HRSEM Quanta FEG, 250 FEI Company). The untreated cellulosic fibers (bamboo, modal and cotton) were also examined as blank samples. Additionally, the elemental analysis and composition were measured by using the energy dispersive X-ray analyzer (EDX, AME-TEK analyzer) which attached with the microscope. X-ray diffractions for the untreated cellulosic fibers and the prepared hybrids (ZIF-8@bamboo, ZIF-8@modal and ZIF-8@cotton) were measured by using Philips X'Pert MPD diffractometer (λ = 1.5406 Å). The infrared spectra were measured for the untreated cellulosic fibers, the synthesized hybrids (ZIF-8@bamboo, ZIF-8@modal and ZIF-8@cotton) and the synthesized hybrids after adsorption of Chlorophyll-a. The attenuated total reflection–Fourier transform infrared spectroscopy (ATR-FTIR) were performed by using JASCO FT/IR-4700 spectrophotometer, (Japan). The absorbance spectra were collected in the wavenumber range of 400–4000 cm^–1^ with 1.0 cm^−1^ interval and repetitive scans of 64. The stability of MOFs within the treated fibers via analyzing surface area and metal content.

### Chlorophyll-a separation

Separation of Chlorophyll-a from spinach extract was systematically studied by adsorption onto the untreated fibers (bamboo, modal and cotton) and the synthesized hybrids (ZIF-8@bamboo, ZIF-8@modal and ZIF-8@cotton). Selectivity of ZIF-8@cellulosic fibers was approved via HPLC analysis, whereas, Analysis was carried out in an Agilent 1100 Model HPLC instrument (Agilent Technologies, Santa Clara, CA, USA) and a UV–*Vis* detector (Santa Clara, CA, USA). The total run time of the analysis was 20 min. The flow rate was 0.90 mL min^−1^, and 20.00 µL of the sample were injected into the HPLC system. Compounds present in the elution were monitored at 450 nm using a UV–*vis* detector. Peaks were identified by their retention time, and were compared to the authentic standard of chlorophyll-a.

Adsorption experiments of Chlorophyll-a were carried out in 15 mL glass flask under different experimental conditions including reaction time and concentration. In the adsorption experiments, 30 mg of the adsorbent (fibers or hybrids) was immersed in 10 mL of chlorophyll extract (2 mg/L) and the mixture was stirred at 30 ± 3 °C for up to 8 h. At the end of experiment, the mixture was centrifuged, filtered and Chlorophyll-a concentration was evaluated by using UV–Visible spectrophotometer (UV-160A, Shimadzu, Japan) and the capacity of adsorption was subsequently estimated. The absorbance of Chlorophyll-a was recorded at λ = 663 nm and the concentration of Chlorophyll-a solution before and after the adsorption were detected by the peak area and standard curve. To study the adsorption kinetics, samples were periodically withdrawn from the reaction at different intervals. In case of study the isotherm, the chlorophyll concentration in the extract was used in the range of 1–9 mg/L and the experiments were performed at 30 ± 3 °C for 1 h. For each experiment, three independent replicated were carried out and the average values were conducted.

### Desorption and recyclability

Chlorophyll release was investigated herein through removal of chlorophyll from the applied adsorbents as desorption process. After the adsorption of chlorophyll, the applied adsorbents (untreated cellulosic fibers and ZIF-8@cellulosic fibers hybrids) were transported to 100 mL flask containing 50 mL from acetone (80%) and then the flask was located for 6 h in the shaking water bath at 70 rpm. The total concentration of Chlorophyll-a inside the solution was evaluated by UV–vis spectrophotometer (UV-160A, Shimadzu, Japan). The kinetic study of Chlorophyll-a release was illustrated in order to optimize the best desorption conditions. Subsequently, the recyclability of the applied materials in Chlorophyll-a separation from spinach extract was performed via investigation the adsorption/desorption. After Chlorophyll-a desorption, the renovated adsorbents were applied in the next adsorption cycle and current process (adsorption/desorption) was consecutively repeated five times. The mechanism of adsorption was approved via elemental analysis, whereas, the percentage calculated from EDS and the results are expressed as mean ± SD for determination of 10 points. C, H, N percentages were measured triplicate.

### Statistical analysis

All adsorption data presented in the current work were conducted in the means for three independent replicates and error bars/standard deviations were included in all data. All parameters of kinetics and isotherms and all presented Figures were obtained and drawn by using Origin 8 program, respectively.

## Conclusion

Separation of Chlorophyll-a from spinach extract by using ZIF-8@cellulosic fibers hybrids was currently investigated. Hybrids were synthesized by directly preparation of ZIF-8 in presence of the cellulosic fibers (bamboo, modal and cotton). Crystalline rods of ZIF-8 were observed over the cellulosic fibers with length of 1.3–4.4 µm. The adsorption of Chlorophyll-a onto the obtained hybrids was fit to pseudo-second order and Langmuir isotherm. The adsorption capacities on to hybrids were followed the order of ZIF-8@cotton > ZIF-8@modal > ZIF-8@bamboo. The maximum capacities were enlarged from 304.2 to 350.2 for untreated cellulosic fibers to 528.7–583.6 mg/g for ZIF-8@cellulosic fibers hybrids. Chlorophyll-a was almost released from hybrids within 6 h. Within 5 repetitive washing cycles, the adsorption capacity of hybrids towards Chlorophyll-a was reduced by 27–28%. It could be hypothesized that, the adsorption of Chlorophyll-a on the hybrids was proceeded via physically with deposition in pores and/or chemically via π–π and electrostatic interaction. The obtained results showed that, the most effective separation of Chlorophyll-a could be performed via the exploitation of ZIF-8@bamboo even after five washing cycles. As analogous with literature, the synthesized hybrids exhibited several advantages represented in the remarkable capacity towards chlorophyll-and recyclability. 

### Supplementary Information

Supplementary Information.

## Data Availability

The all data generated or analyzed during the current study are included in this manuscript.
