# Supplementary material for: Selective separation of chlorophyll-a using recyclable hybrids based on Zn-MOF@cellulosic fibers
Source: Sci Rep. 2023 Sep 14;13:15208. doi: 10.1038/s41598-023-42151-9 (PMC10502031; doi:10.1038/s41598-023-42151-9)
Supplement: Supplementary file 1 — Supplementary Information. [file 41598_2023_42151_MOESM1_ESM.docx]

**Supporting information**

**Figure S1:** HPLC analysis for; **[a]** the spinach extract and **[b]** spinach extract after adsorption of chlorophyll-a.

**
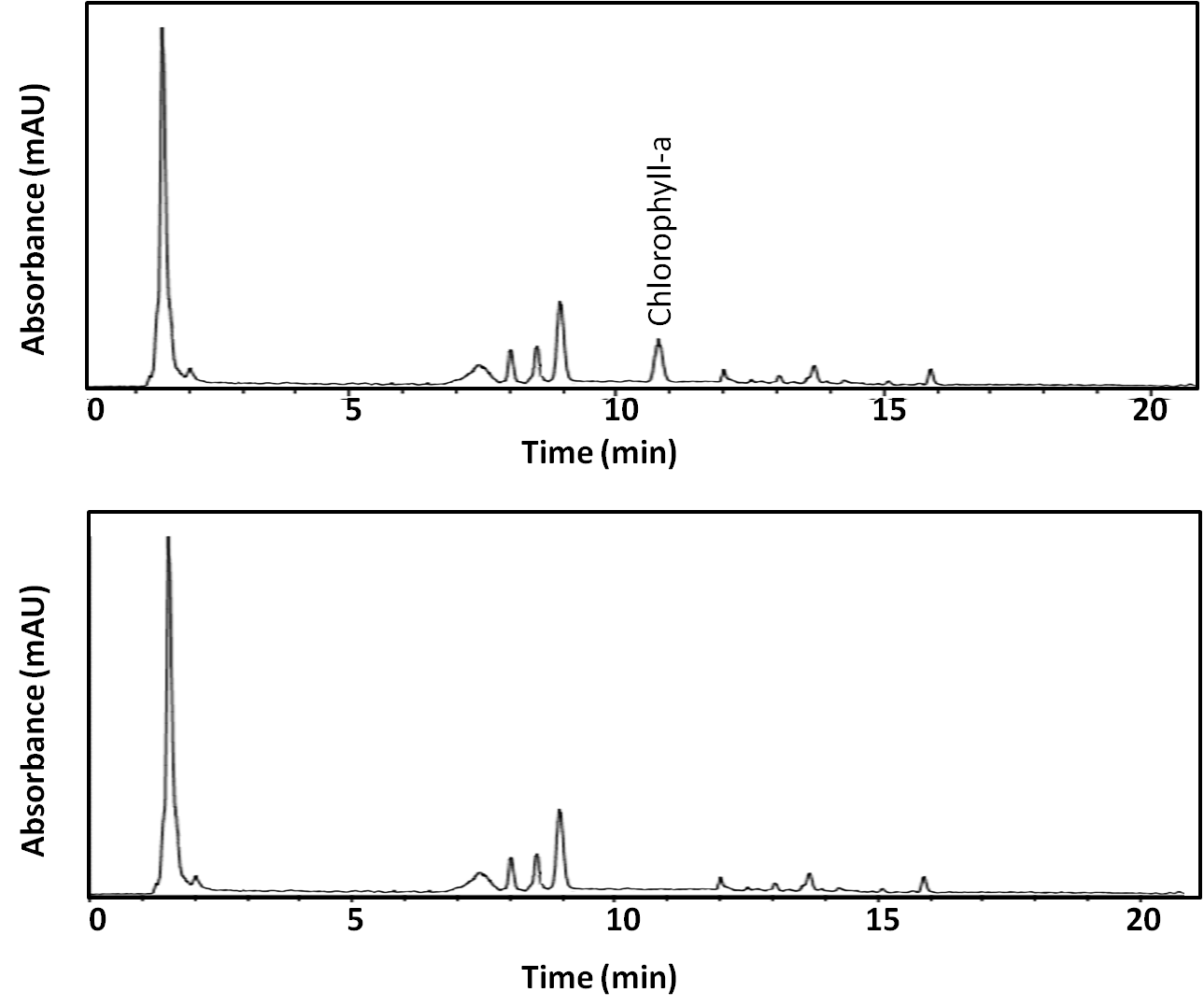
**

**Table S1:** Elemental analysis for the hybrids before and after chlorophyll-a adsorption.

| **Samples** | **Metal ratio*** | | **C** | **H** | **N** |
| --- | --- | --- | --- | --- | --- |
|  | **Zn** | **Mg** |  |  |  |
|  | **Before adsorption** | | | | |
| **ZIF-8@bamboo** | *7.4*5 ± 1.12 | *-* | *43.77* ± 2.43 | *5.97* ± 0.45 | *6.38* ± 0.75 |
| **ZIF-8@modal** | 7.33 ± 0.68 | - | 42.98 ± 1.84 | 6.11 ± 0.48 | 6.12 ± 0.94 |
| **ZIF-8@cotton** | 8.51 ± 0.92 | - | 43.18 ± 2.53 | 6.02 ± 0.72 | 6.19 ± 1.02 |
|  | **After adorption** | | | | |
| **ZIF-8@bamboo** | *1.22* ± 0.27 | *2.27* ± 0.41 | *68.98* ± 2.04 | *7.77* ± 0.92 | *6.29* ± 0.63 |
| **ZIF-8@modal** | 1.32 ± 0.21 | 2.41 ± 0.34 | 67.1 ± 1.46 | 7.07 ± 1.10 | 6.02 ± 0.84 |
| **ZIF-8@cotton** | 2.28 ± 0.33 | 3.32 ± 0.27 | 68.25 ± 1.83 | 7.58 ± 1.13 | 6.31 ± 0.22 |

*the percentage calculated from EDS and the results are expressed as mean ±SD for determination of 10 points. C, H, N percentages were measured triplicate.

**Table S2:** Surface area and metal contents before and after chlorophyll-a adsorption.

|  | **Samples** | **Adsorption cycles** | | | | |
| --- | --- | --- | --- | --- | --- | --- |
|  |  | **Intial** | **1** | **2** | **3** | **4** |
| **Surface area (m^2^/g)** | **ZIF-8@bamboo** | 540 | 510 | 485 | 470 | 458 |
|  | **ZIF-8@modal** | 570 | 563 | 552 | 548 | 539 |
|  | **ZIF-8@cotton** | 610 | 588 | 574 | 567 | 558 |
| **Metal content (mg/g)** | **ZIF-8@bamboo** | *7.45* | 7.38 | 7.33 | 7.21 | 7.18 |
|  | **ZIF-8@modal** | 7.33 | 7.28 | 7.15 | 7.08 | 7.02 |
|  | **ZIF-8@cotton** | 8.51 | 7.41 | 7.34 | 7.25 | 7.19 |

**Figure S2:** UV-Vis spectra for the desorbed chlorophyll-a.
